# Transmission cluster of COVID-19 cases from Uruguay: emergence and spreading of a novel SARS-CoV-2 ORF6 deletion

**DOI:** 10.1590/0074-02760210275

**Published:** 2022-01-10

**Authors:** Yanina Panzera, Natalia Ramos, Lucía Calleros, Ana Marandino, Gonzalo Tomás, Claudia Techera, Sofía Grecco, Sandra Frabasile, Eddie Fuques, Leticia Coppola, Natalia Goñi, Viviana Ramas, Cecilia Sorhouet, Victoria Bormida, Analía Burgueño, María Brasesco, Maria Rosa Garland, Sylvia Molinari, Maria Teresa Perez, Rosina Somma, Silvana Somma, Maria Noelia Morel, Cristina Mogdasy, Héctor Chiparelli, Juan Arbiza, Adriana Delfraro, Ruben Pérez

**Affiliations:** 1 Universidad de la República, Facultad de Ciencias, Instituto de Biología, Departamento de Biología Animal, Sección Genética Evolutiva, Montevideo, Uruguay; 2 Universidad de la República, Facultad de Ciencias, Instituto de Biología e Instituto de Química Biológica, Sección Virología, Montevideo, Uruguay; 3 Ministerio de Salud Pública, Centro Nacional de Referencia de Influenza y Otros Virus Respiratorios, Departamento de Laboratorios de Salud Pública, Montevideo, Uruguay

**Keywords:** genetics, indels, accessory gene, repetitive sequence, coronavirus

## Abstract

**BACKGROUND:**

Evolutionary changes in severe acute respiratory syndrome coronavirus 2 (SARS-CoV-2) include indels in non-structural, structural, and accessory open reading frames (ORFs) or genes.

**OBJECTIVES:**

We track indels in accessory ORFs to infer evolutionary gene patterns and epidemiological links between outbreaks.

**METHODS:**

Genomes from Coronavirus disease 2019 (COVID-19) case-patients were Illumina sequenced using ARTIC_V3. The assembled genomes were analysed to detect substitutions and indels.

**FINDINGS:**

We reported the emergence and spread of a unique 4-nucleotide deletion in the accessory ORF6, an interesting gene with immune modulation activity. The deletion in ORF6 removes one repeat unit of a two 4-nucleotide repeat, which shows that directly repeated sequences in the SARS-CoV-2 genome are associated with indels, even outside the context of extended repeat regions. The 4-nucleotide deletion produces a frameshifting change that results in a protein with two inserted amino acids, increasing the coding information of this accessory ORF. Epidemiological and genomic data indicate that the deletion variant has a single common ancestor and was initially detected in a health care outbreak and later in other COVID-19 cases, establishing a transmission cluster in the Uruguayan population.

**MAIN CONCLUSIONS:**

Our findings provide evidence for the origin and spread of deletion variants and emphasise indels’ importance in epidemiological studies, including differentiating consecutive outbreaks occurring in the same health facility.

Human severe acute respiratory syndrome coronavirus 2 (SARS-CoV-2) is a novel member of the genus *Betacoronavirus* (subgenus *Sarbecovirus*) that causes the pandemic coronavirus disease 2019 (COVID-19).^1^

Like other coronaviruses, SARS-CoV-2 has a relatively large RNA genome (~ 30 kb) with well-characterised open reading frames (ORFs) coding for proteins involved in replication and transcription of the viral genome (nsp1-16) and the structure of the virion (spike, matrix, small envelope, and nucleocapsid). The genome also contains ORFs that code for accessory proteins (3a, 6, 7a, 7b, 8, and 10) that are not directly required for virus viability in cell culture but interfere with cellular processes or modulate the infection process in the natural host.^2^ ^,^ ^3^

The coronavirus genome has high plasticity, as evidenced by the fast evolution driven by point mutations, deletions and insertions (indels), and recombination. This feature contributes to the ongoing rapid transmission and global spread of SARS-CoV-2 in humans and animals and the emergence of strains with new biological properties.^4^

Examining the entire repertoire of genetic variants is crucial to understand better the evolutionary, pathogenic, and antigenic potential of SARS-CoV-2^5^ ^,^ ^6^ ^,^ ^7^ and to detect changes in primer and probe binding sites that affect the sensitivity of diagnostic tests.^8^ In addition, identifying mutations is acquiring more relevance with the emergence of genetic variants of concern that potentially impact virus control and vaccine development.^9^ ^,^ ^10^ ^,^ ^11^

The most common changes in SARS-CoV-2 are single nucleotide polymorphisms (SNPs),^12^ ^,^ ^13^ but indels are also detected at high frequency. Indels evidence another aspect of genomic evolution and provide additional data about the virus’s capability to maintain its functionality regardless of changes in genome size. Indels are also valuable and stable markers to use in conjunction with SNPs to establish and characterise transmission clusters (i.e., groups of individuals infected with similar or identical variants that derive from a recent common ancestor). Short indels are unevenly distributed in the SARS-CoV-2 genome and are particularly common in the S gene that encodes the spike glycoprotein, the virus surface protein that determines the infectivity and host range of coronaviruses.^14^ ^,^ ^15^ Some of these indels might increase the variability of the S gene and help adaptation to the human host. Indels are also common in other structural and non-structural coding regions (N, E, ORF1ab).^16^ ^,^ ^17^ ^,^ ^18^ Accessory genes also harbor indels, particularly in the ORFs 3a, 6, 7a, and 8.^19^ ^,^ ^20^ ^,^ ^21^ ^,^ ^22^ ^,^ ^23^

Tracking indels in accessory ORFs is interesting from an evolutionary perspective and may also provide clues about their functionality.^24^ Furthermore, given the potential involvement in pathogenesis and virulence, indels that alter protein function may represent the ongoing viral adaptation to humans through natural attenuation.^25^ ^,^ ^26^

The present study identified and described a 4-nucleotide deletion in the ORF6 coding for protein 6 (p6). The 61-amino acids p6 plays a critical role in viral replication, counteracting host antiviral response, and inhibiting type I interferon production and downstream signaling.^26^ ^,^ ^27^ Phylogenetic analysis and deletion presence allow identifying a transmission cluster of COVID-19 cases from Uruguay and provide insights into the origin and spreading of gene deletions in SARS-CoV-2.

## MATERIALS AND METHODS

Eleven COVID-19 case-patients were diagnosed as part of the ongoing laboratory surveillance at the National Reference Centre for Influenza and other Respiratory Viruses (DLSP-MSP, Uruguay). Five cases had an epidemiologic link and were diagnosed between September 28th and October 6th (2020) associated with an outbreak in a health care centre. The second group of cases did not share a clear epidemiological nexus and were diagnosed from October 1st to November 10th (2020) (Supplementary data - Table I).

Combined nasopharyngeal and oropharyngeal swab samples were obtained, and the diagnosis was performed by RNA extraction (Qiamp Viral RNA Minikit, Qiagen USA) followed by real-time reverse transcription-polymerase chain reaction (RT-qPCR) using the protocol recommended by the Panamerican Health Organization (PAHO-WHO).^28^ SARS-CoV-2 RNA samples were then submitted to the Genomic Platform at the Faculty of Science, University of the Republic, Uruguay (UdelaR), for genome sequencing and analysis.

Multiplex PCR was performed using ARTIC primer scheme version 3; primer sequences and protocols are available at the ARTIC network repository (https://artic.network/ncov-2019). Complementary DNA (cDNA) and Nextera DNA Flex library preparation were performed following previously published conditions.^22^ Whole-genome sequencing was performed on an Illumina MiniSeq (Illumina, USA) platform using MiniSeq^TM^ Mid Output Reagent Cartridge (300-cycles, paired-end reads). Adapter/quality trimming and filtering raw data were performed with BBDuk, and clean reads were mapped to the consensus genome using Geneious Prime 2020.1.2 (https://www.geneious.com). Complete genomes with broad average coverage (> 200) were obtained, annotated, and submitted to the GenBank.

The deletion was confirmed using RT-PCR with ARTIC primers flanking the deleted region (forward: TCTTGCTTTGCTGCTGCTTG, reverse: TGAAATGGTGAATTGCCCTCGT) and further Sanger sequencing of the generated amplicon (~ 370 bp) in Macrogen (Korea).

The online web application CoV-GLUE (http://cov-glue.cvr.gla.ac.uk/#/deletion) was used to assess amino acid replacement and indels in SARS-CoV-2 genomes and to analyse the frequency and geographic location of detected deletions.^29^ Lineage was assigned according to the nomenclature system proposed by Rambaut et al.^30^ using the Pangolin COVID-19 Lineage Assigner server (https://pangolin.cog-uk.io/).

All B.1.1.33 and N.7 lineages sequences (about 2000 sequences) were retrieved from the GISAD EpiCoV database^31^ and the GenBank database to create the dataset for phylogenetic analysis. Multiple alignments were performed with MAFFT.^32^ With a 1000-replicate bootstrap to support internal nodes, maximum-likelihood trees were inferred in Geneious using the FastTree plugin^33^ and visualised with the ggTree package in R.

The structure model of the ORF6 protein was obtained from the I-TASSER server (https://zhanglab.ccmb.med.umich.edu/COVID-19/).

## RESULTS

**Fig. 1:**
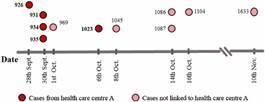
schematic timeline of the transmission cluster analysed in this study. The timeframe spanned 28th September to 10th November 2020. Darker red dots correspond to cases from health care centre A, and lighter red dots correspond to cases with no documented epidemiological link to health care centre A. Epidemiological data of the samples is available in Supplementary data (Table I).

**Fig. 2:**
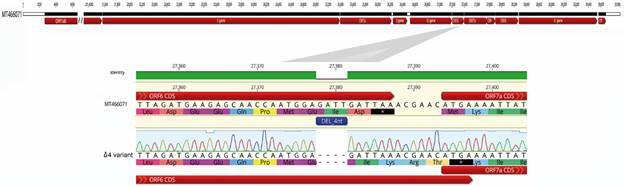
top: complete severe acute respiratory syndrome coronavirus 2 (SARS-CoV-2) genome organisation is schematised. The Mdeo-1 sequence (MT466071) was used as a reference. Bottom: zoomed diagram to show the location of the deletions in the ORF6 detailing nucleotide deletion and amino acid changes. Chromatogram peaks of the Δ4 variant showed the validation by Sanger sequencing.

*Detection of deletions and Sanger characterisation* - We identified a novel 4-nucleotide deletion (Δ4) in eleven SARS-CoV-2 genomes from Uruguayan patients collected from September to November 2020 (Fig. 1). The Δ4 change is located at 27378-27381 position in the accessory ORF6 and constitutes a frameshifting change (i.e., a mutation that shift the reading frame used for translation of the protein) that was found in 100% of the reads in all eleven full-length-genomes and Sanger sequences (Fig. 2).

The frameshifting deletion (GATT) involves the last Aspartate codon (GAT) and the first base of the stop codon (TAA) of the ORF6. It extends the reading frame to a new stop codon (TGA) located seven nucleotides downstream. Therefore, the deletion produces a substitution of the last amino acid (Asp→Lys) and increases the coding region of the ORF6 by two amino acids (Arg, Thr) at the end of the 61-amino acid protein, resulting in a putative protein of 63 amino acids. The new stop codon (TAA → TGA) overlaps with the initial codon of the following ORF7a (Fig. 2).

**Fig. 3:**
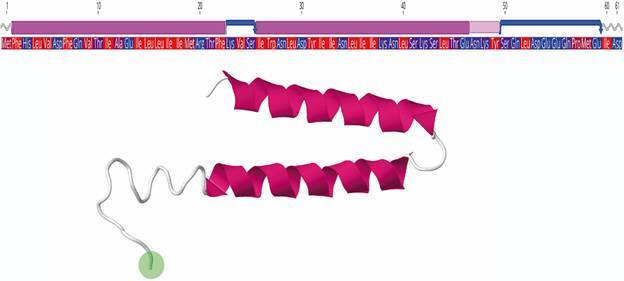
structure of the 61-amino acids ORF6 protein (p6) of severe acute respiratory syndrome coronavirus 2 (SARS-CoV-2). Top: amino acid sequence and functional protein domains and folds. Pink cylinders represent α-helices, and blue arrows denote ß-strands. Bottom: three-dimensional model. The green circle denotes the deletion region detected in Uruguayan samples.

The I-TASSER theoretical model of p6 is structured as two α-helices separated by a flexible random coil or turn (Fig. 3). The first helix begins at Phe2 and extends to Phe22, while the second helix is predicted to begin at Ile26 and extend to Glu44; a more disordered region extends from Ser50 to Asp61 (the last two residues are structured in a coil). The deletion here described is located in the disordered coil region and is part of a larger functional domain (ten last residues of the disordered region) involved in nuclear import obstruction in the homologous p6 in SARS-CoV-1.^34^

*Lineage classification and phylogenetic analysis of complete genomes* - The complete eleven genomes of the Δ4 variant were classified as belonging to the SARS-CoV-2 phylogenetic lineage B.1.1.33 using the Pangolin web application.

**Fig. 4:**
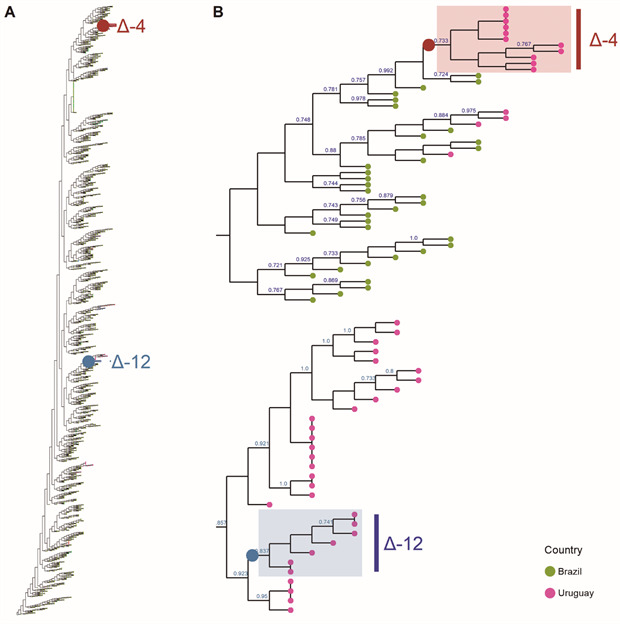
maximum likelihood phylogenetic analysis including the Δ4 and the previously reported Δ12 sequences from Uruguay, together with B.1.1.33 and B.1.1.33.7 (or N.7) sequences available at GISAID from South America (A). Subclades including Δ4 and Δ12 sequences are depicted in detail (B). The most related to the Δ4 sequences reported in this paper are B.1.1.33 sequences from Brazil. A large version of the phylogenetic tree with labeled tips is available in Supplementary data (Fig. 1).

Phylogenetic analysis revealed that the Δ4 variant clustered with six Brazilian strains from Rio Grande do Sul, the southernmost state of Brazil [Fig. 4, Supplementary data (Fig. 1)]. These six Brazilian sequences have four unassigned bases (NNNNs) in the deletion position.

Other Uruguayan strains belonging to the B.1.1.33 lineage fell in a separate clade (Fig. 4). The Δ4 change was not detected in any of the Uruguayan strains available at GISAID nor any South American, European, or Asiatic available sequences in the CoV-GLUE database by May 2021. A single North American sequence has the same deletion (MT520188) but belongs to the phylogenetically unrelated lineage B.1 [Supplementary data (Table II)].

*Comparison with a previous outbreak in the same health care centre* - Sequences were compared with those of a previous outbreak from the same care centre (health care centre A in this study), where the five initial cases of the Δ4 variant were detected (Fig. 1). The first outbreak corresponded to the Uruguayan B.1.1.33.7 lineage, also known as N.7, with a 12-nucleotide deletion (Δ12) in the ORF7a.^22^ The second outbreak comprised only viruses from the B.1.1.33 lineage with the 4-nucleotide deletion (Δ4 variant). Both lineages appear widely separated in the phylogenetic tree [Fig. 4, Supplementary data (Fig. 1)]. Besides, unlike deletions in different genes, they have 17 SNPs, including ten non-synonymous changes affecting ORF1ab (six residues), ORF8 (one residue), and N (two residues).

## DISCUSSION

The present study identified a novel Δ4 variant that extends the reading frame to a new stop codon (TGA) and then increases the coding region of the ORF6 by two amino acids (Arg, Thr), resulting in a putative protein of 63 residues. The new stop codon (TAA → TGA) overlaps with the initial codon of the following ORF7a (Fig. 2). The most used stop codon in SARS-CoV-2 is TAA, the most efficient stop codon in humans, being used by highly expressed proteins in the lung cells.^35^ However, TGA is associated with functional read-through phenomena in humans.^36^ In other viruses, it has been proposed that codon usage bias that mimics those of the highly expressed genes in the host has implications for pathogenesis, given the translational competition for tRNAs.^37^ ^,^ ^38^ The change observed here from a TAA to a TGA stop may indicate a less adapted codon usage^35^ ^,^ ^39^ ^,^ ^40^ or a quasi-neutral alternative evolutionary path for accessory gene divergence.

Deletions of diverse size and prevalence have been described in the ORF6 [Supplementary data (Table II, Fig. 2)]. A 27 nucleotide in-frame deletion emerged during passaging in cell culture, possibly due to the *in vitro* removal of selective pressure eliminating nine amino acids near the centre of the protein.^41^ Two frameshifting deletions of 26 and 34 nucleotides resulted in truncated proteins.^23^ An identical 4-nucleotide deletion to the one reported occurs in a single sequence from the United States (Accession number: MT520188), but mutation was described as an insertion because the analysis was performed using protein sequences.^42^

Several deletions are likely random and sporadic events that lead the virus to a loss in coding information that might alter protein function and thus generate a potential reduction in fitness.^20^ However, in relatively few cases, the deletion persists and becomes a circulating form that successfully spreads in the population. The Δ4 change is remarkable because it is a gene deletion that increases p6 coding information that does not seem to affect the viral transmission, converting the Δ4 variant into a SARS-CoV-2 circulating form in the Uruguayan population.

SARS-CoV-2 p6 binds karyopherins (KPNA1 and KPNB1) and leads to a disruption of the nucleocytoplasmic transport, preventing the nuclear translocation of STAT1 (signal transducer and activator of transcription 1) in response to interferon signaling. It also interferes with STAT1 translocation by binding to Nup98, a crucial component of nuclear pore complexes.^43^ This disruption ultimately blocks the expression of interferon-stimulated genes (ISGs) that display multiple antiviral activities and play a fundamental role in the pathogeny of COVID-19.^43^ ^,^ ^44^

The effect of deletions on protein functionality is difficult to determine, especially when there is no crystallographic structure of the protein, as is the case of p6. The I-TASSER theoretical model of p6 is structured as two α-helices separated by a flexible random coil or turn (Fig. 3). The first helix begins at Phe2 and extends to Phe22, while the second helix is predicted to begin at Ile26 and extend to Glu44; a more disordered region extends from Ser50 to Asp61 (the last two residues are structured in a coil). The deletion here described is located in the disordered coil region and is part of a larger functional domain (ten last residues of the disordered region) involved in nuclear import obstruction in the homologous p6 in SARS-CoV-1.^34^ This functional domain is critical for karyopherins binding and STAT1 blockage.^45^ A recent study indicates that the C-terminal tail of p6 (positions 53 - 61) is essential for inhibiting IFN-I production through the interaction with IRF3 and STAT-1 proteins, whereas amino acids 49 to 52 are dispensable.^26^ This study also suggests that the C-terminal residues in p6 from SARS-Cov-1 and 2 were enriched in negative-charged amino acids, promoting their interaction with cellular proteins and thus favoring its antagonistic activity.^26^ The Δ4 deletion found in the sequences reported here results in a C-terminus bearing positive charged (Lys 61, Arg 62) and neutral (Thr 63) amino acids [Fig. 3, Supplementary data (Fig. 3)]. Based on these data and our results, the Δ4 deletion may produce subtle changes in p6 that modify the ability of SARS-CoV-2 to evade the host’s innate immune system.

The complete eleven genomes of the Δ4 variant were classified as belonging to the B.1.1.33 lineage that emerged in Brazil and spread in several South American countries during the early pandemic phase.^46^ ^,^ ^47^ This lineage is defined by two non-synonymous substitutions in ORF6 (T27299C/I33T) and the nucleocapsid protein (T29148C/I292T), markers that are also strongly conserved in all Uruguayan strains of this lineage.

The high nucleotide identity of the eleven Δ4 variant genomes (seven sequences were identical, two have 1 SNP, and the remaining two have 2 SNPs), the close phylogenetic relationship between them (Fig. 4), and the epidemiological data (Fig. 1) strongly suggest that the Δ4 variant emerged once from a recent B.1.1.33 ancestor [Supplementary data (Fig. 1)]. Thus, the emergence and spreading of the Δ4 variant (initial cases from a defined outbreak and later cases without a confirmed epidemiological link) established a transmission cluster in the Uruguayan population.

Notably, the six Brazilian sequences more closely related to the Δ4 variant have unassigned bases (four Ns track) in the deletion position, and we cannot discard that they were deleted strains.

The same deletion observed in the Δ4 Uruguayan variant was also observed in the North American MT520188 strain that belongs to a different lineage (B.1), indicating that this 4-nucleotide emerged independently in the United States. The occurrence of the same deletion in phylogenetically unrelated sequences suggests a common molecular mechanism for its origin. In other organisms, indels occur preferentially at homo-polymeric tracts and short repeated sequences.^48^ Repeated sequences may induce replication slippage, in which the template strand and its copy shift their relative positions so that part of the template is either copied twice or missed out. The Δ4 alteration deletes one repeat unit (GATT) of a two 4-nucleotide repeat (GATT-GATT). Our result shows that deletion of directly repeated sequences also occurs in the SARS-CoV-2 genome, even outside the context of extended repeat regions (n = 2). Accordingly, the repeated region might represent a putative initiator of an indel event in the SARS-CoV-2 genome.

A previous report identified a 12-nt deletion (Δ12) in the ORF7a detected two months earlier in the same health care centre as the Δ4 variant.^22^ However, these two outbreaks do not belong to the same transmission cluster because, aside from different deletions (Δ12 and A4), sequences belong to different phylogenetic clades (N.7 and B.1.1.33) that differ in more than 14 SNPs [Supplementary data (Fig. 1)].

This finding indicates that successive outbreaks in this care centre were caused by infection of a new lineage and not by the persistence of viruses from the initial outbreak.

The detection of two deletions in a small sample of COVID-19 cases in Uruguay is surprising. We hypothesise that during the current epidemic phase in the naïve human host, SARS-CoV-2 did not have intense competition and successfully attempted different variability strategies, including a high frequency of deletions.

The analysis of deletions in the Uruguayan population can track changes in the pandemic course, providing new insight into the evolutionary dynamics of SARS-CoV-2. During the early stages of the pandemic in Uruguay, most cases were restricted to outbreaks associated with social events, workplaces, and health or residential care centres, where the origin and epidemiological connections of the transmission network were readily inferred.^49^ In that context, we described the Δ12 variant restricted to a single outbreak. However, in late 2020, there was a gradual increase in the number of cases of SARS-CoV-2 in Uruguay, leading to an extensive community circulation and the subsequent loss of the epidemiological nexus for a considerable fraction of COVID-19 cases. An early signal of this new epidemiological scenario may be exemplified by the Δ4 variant, which was initially associated with a specific outbreak and later detected in individuals without apparent epidemiological links (Fig. 1). In this new phase, genomic surveillance acquired even more relevance to inferred transmission networks, and indels became robust markers that bring precise information and are easy to analyse. Furthermore, indels provide a fine-scale resolution when the genomes are closely related, as observed in Uruguay with the B.1.1.33 lineage, which included viruses with or without the deletion [Fig. 4, Supplementary data (Fig. 1)].

Our results reinforce the importance of indel analysis to evidence epidemiological changes in the populations and contribute to understanding the origin and spreading of variants in the host population. We emphasise that more epidemiologically helpful deletions are monophyletic and occur in a specific geographic area.

Even though there are hundreds of indels in SARS-CoV-2, deletions in repeat regions like the here described in the ORF6 provide new data about the molecular origin of indels and deserve our special attention. Moreover, deletions with a known spreading pattern are uncommon and relevant to establishing the origin of reinfections and detecting transmission chains with a high prevalence and genome homogeneity. Integration of high-resolution genomic data, including SNPs and indels, with epidemiolocal data and gene function, is a successful approach to investigate the genome evolution of SARS-CoV-2.

*Ethics* **-** The research described in this study was performed in adherence to the Declaration of Helsinki; no specific authorisation was required because the activities were conducted as part of routine virological surveillance (anonymously, without identification of patients) by the Uruguayan official Institution for surveillance of Influenza and other respiratory viruses of the Ministry of Public Health (DLSP-MSP).

*Data availability* - Viral sequences were deposited in the GenBank (accession numbers: MZ312086‒MZ312096).

## References

[1] Zhou P, Yang X-L, Wang X-G, Hu B, Zhang L, Zhang W (2020). A pneumonia outbreak associated with a new coronavirus of probable bat origin. Nature.

[2] Narayanan K, Huang C, Makino S (2008). SARS coronavirus accessory proteins. Virus Res.

[3] Li JY, Liao CH, Wang Q, Tan YJ, Luo R, Qiu Y (2020). The ORF6, ORF8 and nucleocapsid proteins of SARS-CoV-2 inhibit type I interferon signaling pathway. Virus Res.

[4] Cagliani R, Forni D, Clerici M, Sironi M (2020). Coding potential and sequence conservation of SARS-CoV-2 and related animal viruses. Infect Genet Evol.

[5] Sabino EC, Buss LF, Carvalho MPSS, Prete CA, Crispim MAEE, Fraiji NA (2021). Resurgence of COVID-19 in Manaus, Brazil, despite high seroprevalence. Lancet.

[6] Resende PC, Naveca FG, Lins RD, Dezordi FZ, Ferraz MVF, Moreira EG (2021). The ongoing evolution of variants of concern and interest of SARS-CoV-2 in Brazil revealed by convergent indels in the amino (N)-terminal domain of the Spike protein. Virus Evol.

[7] Wibmer CK, Ayres F, Hermanus T, Madzivhandila M, Kgagudi P, Oosthuysen B (2021). SARS-CoV-2 501Y V2 escapes neutralization by South African COVID-19 donor plasma. Nat Med.

[8] Bal A, Destras G, Gaymard A, Stefic K, Marlet J, Eymieux S (2021). Two-step strategy for the identification of SARS-CoV-2 variant of concern 202012/01 and other variants with spike deletion H69-V70, France, August to December 2020. Eurosurveillance.

[9] Tegally H, Wilkinson E, Giovanetti M, Iranzadeh A, Fonseca V, Giandhari J (2020). Emergence and rapid spread of a new severe acute respiratory syndrome-related coronavirus 2 (SARS-CoV-2) lineage with multiple spike mutations in South Africa. medRxiv.

[10] Faria NR, Claro IM, Candido D, Franco LAM, Andrade PS, Thais M (2021). Genomic characterisation of an emergent SARS-CoV-2 lineage in Manaus preliminary findings. Virological.

[11] Volz E, Mishra S, Chand M, Barrett JC, Johnson R, Geidelberg L (2021). Transmission of SARS-CoV-2 Lineage B.1.1.7 in England: insights from linking epidemiological and genetic data. medRxiv.

[12] Badua CLDC, Baldo KAT, Medina PMB (2020). Genomic and proteomic mutation landscapes of SARS-CoV-2. J Med Virol.

[13] Franco-Muñoz C, Álvarez-Díaz DA, Laiton-Donato K, Wiesner M, Escandón P, Usme-Ciro JA (2020). Substitutions in spike and nucleocapsid proteins of SARS-CoV-2 circulating in South America. Infect Genet Evol.

[14] McCarthy KR, Rennick LJ, Nambulli S, Robinson-McCarthy LR, Bain WG, Haidar G (2021). Natural deletions in the SARS-CoV-2 spike glycoprotein drive antibody escape. Science.

[15] Liu P, Jiang J-Z, Wan X-F, Hua Y, Li L, Zhou J (2020). Are pangolins the intermediate host of the 2019 novel coronavirus (SARS-CoV-2). PLoS Pathog.

[16] Bal A, Destras G, Gaymard A, Bouscambert-Duchamp M, Valette M, Escuret V (2020). Molecular characterization of SARS-CoV-2 in the first COVID-19 cluster in France reveals an amino acid deletion in nsp2 (Asp268del). Clin Microbiol Infect.

[17] Benedetti F, Snyder GA, Giovanetti M, Angeletti S, Gallo RC, Ciccozzi M (2020). Emerging of a SARS-CoV-2 viral strain with a deletion in nsp1. J Transl Med.

[18] Kumar BK, Rohit A, Prithvisagar KS, Rai P, Karunasagar I, Karunasagar I (2020). Deletion in the C-terminal region of the envelope glycoprotein in some of the Indian SARS-CoV-2 genome. Virus Res.

[19] Holland LA, Kaelin EA, Maqsood R, Estifanos B, Wu LI, Varsani A (2020). An 81 nucleotide deletion in SARS-CoV-2 ORF7a identified from sentinel surveillance in Arizona (Jan-Mar 2020). J Virol.

[20] Su YCF, Anderson DE, Young BE, Linster M, Zhu F, Jayakumar J (2020). Discovery and genomic characterization of a 382-nucleotide deletion in ORF7b and ORF8 during the early evolution of SARS-CoV-2. MBio.

[21] Gong Y-N, Tsao K-C, Hsiao M-J, Huang C-G, Huang P-WP-N, Huang P-WP-N (2020). SARS-CoV-2 genomic surveillance in Taiwan revealed novel ORF8-deletion mutant and clade possibly associated with infections in Middle East. Emerg Microbes Infect.

[22] Panzera Y, Ramos N, Frabasile S, Calleros L, Marandino A, Tomás G (2021). A deletion in SARS-CoV-2 ORF7 identified in COVID-19 outbreak in Uruguay. Transbound Emerg Dis.

[23] Quéromès G, Destras G, Bal A, Regue H, Burfin G, Brun S (2021). Characterization of SARS-CoV-2 ORF6 deletion variants detected in a nosocomial cluster during routine genomic surveillance, Lyon, France. Emerg Microbes Infect.

[24] Zinzula L (2020). Lost in deletion the enigmatic ORF8 protein of SARS-CoV-2. Biochem Biophys Res Commun.

[25] Lau S-Y, Wang P, Mok BW-Y, Zhang AJ, Chu H, Lee AC-Y (2020). Attenuated SARS-CoV-2 variants with deletions at the S1/S2 junction. Emerg Microbes Infect.

[26] Lei X, Dong X, Ma R, Wang W, Xiao X, Tian Z (2020). Activation and evasion of type I interferon responses by SARS-CoV-2. Nat Commun.

[27] Yuen CK, Lam JY, Wong WM, Mak LF, Wang X, Chu H (2020). SARS-CoV-2 nsp13, nsp14, nsp15 and orf6 function as potent interferon antagonists. Emerg Microbes Infect.

[28] Corman VM, Landt O, Kaiser M, Molenkamp R, Meijer A, Chu DKW (2020). Detection of 2019 novel coronavirus (2019-nCoV) by real-time RT-PCR. Euro Surveill.

[29] Singer J, Gifford R, Cotten M, Robertson D (2020). CoV-GLUE: a web application for tracking SARS-CoV-2 genomic variation. preprints.org.

[30] Rambaut A, Holmes EC, O'Toole Á.Hill V.McCrone JT.Ruis C (2020). A dynamic nomenclature proposal for SARS-CoV-2 lineages to assist genomic epidemiology. Nat Microbiol.

[31] Shu Y, McCauley J (2017). GISAID global initiative on sharing all influenza data - from vision to reality. Euro Surveill.

[32] Katoh K, Standley DM (2013). MAFFT Multiple Sequence Alignment Software Version 7 improvements in performance and usability. Mol Biol Evol.

[33] Price MN, Dehal PS, Arkin AP, Liu DX, Fung TS, Chong KK (2009). FastTree computing large minimum evolution trees with profiles instead of a distance matrix. Mol Biol Evol.

[34] Hussain S, Gallagher T (2010). SARS-coronavirus protein 6 conformations required to impede protein import into the nucleus. Virus Res.

[35] Maldonado LL, Bertelli AM, Kamenetzky L (2021). Molecular features similarities between SARS-CoV-2, SARS, MERS and key human genes could favour the viral infections and trigger collateral effects. Sci Rep.

[36] Seoighe C, Kiniry SJ, Peters A, Baranov PV, Yang H (2020). Selection shapes synonymous stop codon use in mammals. J Mol Evol.

[37] Bahir I, Fromer M, Prat Y, Linial M (2009). Viral adaptation to host a proteome-based analysis of codon usage and amino acid preferences. Mol Syst Biol.

[38] Cheng S, Wu H, Chen Z (2020). Evolution of transmissible gastroenteritis virus (TGEV) a codon usage perspective. Int J Mol Sci.

[39] Nambou K, Anakpa M (2020). Deciphering the co-adaptation of codon usage between respiratory coronaviruses and their human host uncovers candidate therapeutics for COVID-19. Infect Genet Evol.

[40] Huang W, Guo Y, Li N, Feng Y, Xiao L (2021). Codon usage analysis of zoonotic coronaviruses reveals lower adaptation to humans by SARS-CoV-2. Infect Genet Evol.

[41] Riojas M, Frank A, Puthuveetil N, Flores B, Parker M, King S (2020). A rare deletion in SARS-CoV-2 ORF6 dramatically alters the predicted three-dimensional structure of the resultant protein. bioRxiv.

[42] Nguyen TT, Pathirana PN, Nguyen T, Nguyen QVH, Bhatti A, Nguyen DC (2021). Genomic mutations and changes in protein secondary structure and solvent accessibility of SARS-CoV-2 (COVID-19 virus). Sci Rep.

[43] Miorin L, Kehrer T, Sanchez-Aparicio MT, Zhang K, Cohen P, Patel RS (2020). SARS-CoV-2 Orf6 hijacks Nup98 to block STAT nuclear import and antagonize interferon signaling. Proc Natl Acad Sci.

[44] Matsuyama T, Kubli SP, Yoshinaga SK, Pfeffer K, Mak TW (2020). An aberrant STAT pathway is central to COVID-19. Cell Death Differ.

[45] Frieman M, Yount B, Heise M, Kopecky-Bromberg SA, Palese P, Baric RS (2007). Severe acute respiratory syndrome coronavirus ORF6 antagonizes STAT1 function by sequestering nuclear import factors on the rough endoplasmic eeticulum/Golgi membrane. J Virol.

[46] Resende PC, Delatorre E, Gräf T, Mir D, Motta FC, Appolinario LR (2020). Evolutionary dynamics and dissemination pattern of the SARS-CoV-2 lineage B 1.1.33 during the early pandemic phase in Brazil. Front Microbiol.

[47] Candido DS, Claro IM, de Jesus JG, Souza WM, Moreira FRR, Dellicour S (2020). Evolution and epidemic spread of SARS-CoV-2 in Brazil. Science.

[48] Domingo E (2020). Molecular basis of genetic variation of viruses. Elsevier.

[49] Ministerio de Salud Pública (2020). Informe epidemiológico COVID-19 del 6 de noviembre de 2020. https://www.gub.uy/ministerio-salud-publica/comunicacion/noticias/informe-epidemiologico-covid-19-del-6-noviembre-2020.

